# Two-stage Reconstruction of the Large and Ptotic Breasts: Skin Reduction Mastectomy with Prepectoral Device Placement

**DOI:** 10.1097/GOX.0000000000001853

**Published:** 2018-07-12

**Authors:** Charalambos K. Rammos, Denise Mammolito, Victor A. King, Aran Yoo

**Affiliations:** From the Division of Plastic and Reconstructive Surgery, University of Illinois College of Medicine at Peoria, Peoria, Ill.

## Abstract

Wise pattern skin reduction mastectomy with prepectoral placement of the device is a recent technique for reconstruction in patients with large and ptotic breasts. Expanders in the first stage, followed by implant exchange in the second stage are placed above the pectoralis major muscle, totally covered by acellular dermal matrix and an inferior dermal flap. This technique was performed on 6 breasts in 4 obese patients with macromastia and grade 2 and 3 ptosis. Two patients experienced complications at the T-junction. One patient experienced superficial skin sloughing managed conservatively. The second patient developed full-thickness necrosis treated with excision and primary closure. No implant loss occurred. All patients were exchanged in a second stage to an implant, and 2 of them had symmetry procedures, with good cosmetic results. Larger, long-term studies are required to further characterize results and define the limitations of this newer surgical technique.

## INTRODUCTION

Prosthetic reconstruction is the most common reconstructive technique following skin-sparing mastectomy. The traditional approach is the placement of the device in the subpectoral plane. A concern with this approach is the risk of animation deformity and discomfort, due to partial disinsertion of the pectoralis major muscle.^1^ Prepectoral reconstruction resolves many of these issues, by placing the device above the muscle.^2–5^ Patients presenting with large and ptotic breasts remain a challenge, and skin reduction surgery is necessary at the time of reconstruction to achieve an aesthetically pleasing result.

This study describes a newer technique of immediate 2-stage breast reconstruction for the large and ptotic breasts. Skin reduction is performed using the Wise pattern technique while the device is placed in the prepectoral space covered completely by acellular dermal matrix and an inferior dermal flap.

## PATIENTS AND METHODS

An institutional review board–approved retrospective review of prospectively collected data was performed. From October of 2016 to December 2017, this technique was performed in 4 selected patients who required mastectomy and immediate reconstruction. All cases were performed in an academic center. Reconstructive inclusion criteria were large and ptotic breasts with grade 2 or grade 3 ptosis. Need for postoperative radiation was not an exclusive criterion. Demographic, clinical, procedural, and adjuvant treatment data were collected. All postoperative complications were recorded. The charts were reviewed to identify the presence of hematoma, seroma, skin loss, wound infection, device loss, and systematic complications including deep venous thrombosis and pulmonary embolism.

## MATERIALS

All first-stage reconstructions were performed with tissue expanders with suture tabs (Natrelle 133 Tissue Expanders; Allergan, Inc., Irvine, Calif.) and 16 × 20 cm acellular dermal matrix (Alloderm, LifeCell). In the second stage, the expanders were exchanged for smooth cohesive round silicone gel implants (Natrelle Inspira Cohesive; Allergan, Inc., Irvine, Calif.).

### Surgical Technique

**Fig. 1.**
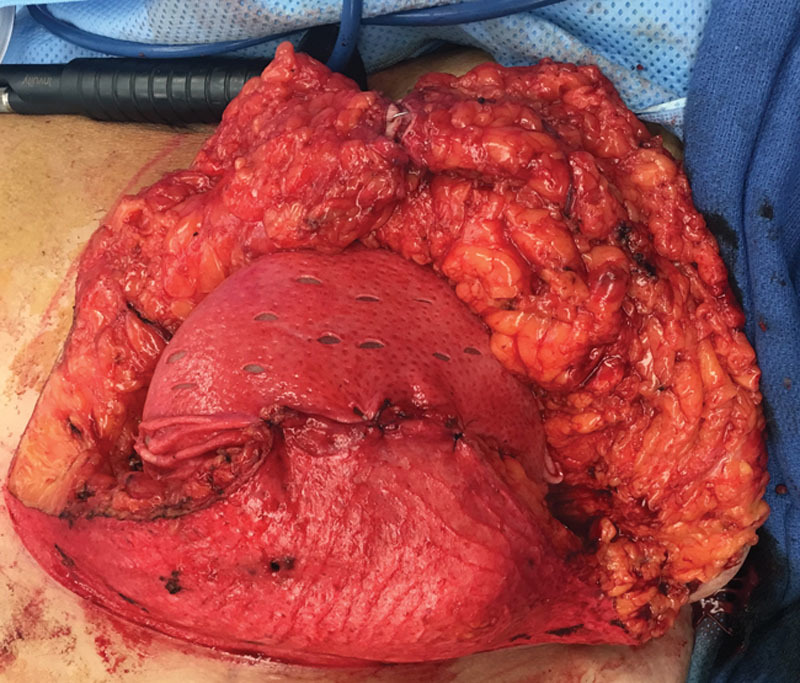
Tissue expander inside the prepectoral pocket, covered completely by acellular dermal matrix and the inferior dermal flap.

Preoperative markings are similar to those for reduction mammaplasty. Because a device will be used, less skin is marked for excision. Additional skin is excised after the device has been placed as necessary. The patient is marked in the upright standing position, and the following markings are made: midline, from sternal notch to xiphoid, inframammary fold, and breast meridian. The new nipple location is marked by transposition of the inframammary fold to the front of the breast, at the level of the meridian. Vertical limbs are drawn from the new nipple location. The length of the vertical limbs is tailored to each patient and for most patients is 10 cm. The distance between the bottom of the vertical limbs is between 10 and 12 cm. The inferior ends of the vertical limbs are then joined to the inframammary fold. Measurements are made bilaterally, from sternal notch to nipple, and from midline to ensure symmetry. A skin-sparing mastectomy is then performed through use of both vertical limbs. After completion of the mastectomy, the expander is wrapped circumferentially with the 16 × 20 cm fenestrated Alloderm sheet and secured to the chest wall in the prepectoral position by suturing the tabs with 2-0 PDS suture. The skin between the inferior ends of the vertical limbs and the inframammary fold is deepithelialized and fenestrated with an 11 blade knife. Inferiorly, the deepithelialized dermal flap is draped over the expander and sutured to the Alloderm superiorly (Fig. 1). The medial and lateral skin flaps are draped to cover the expander, Alloderm, and inferior dermal flap, and approximated to the inframammary fold skin edge inferiorly. Incisions are temporarily stapled, and intraoperative tissue angiography assessment follows, with the use of indocyanine green. Intraoperative expansion is performed with air, and the volume injected is based on the perfusion of the flaps. The wounds are closed in a layered fashion using 3-0 Monocryl suture. Two drains per breast are routinely placed, and patients are admitted at the facility for overnight observation.

Expansion starts approximately in 3 weeks, where the intraoperatively injected air is exchanged for fluid. Expansion then continues on a weekly basis. Underfilling of the tissue expanders relative to final implant placement is preferred as described by Sbitany et al.^6^ This allows for a placement of a larger implant in a tighter breast pocket. Expanders are then exchanged for smooth round highly cohesive implants, during the second stage of the reconstruction. Fat grafting is performed on all reconstructed breasts during implant exchange, using the Revolve device (LifeCell). In cases of no postoperative radiation treatment, the expanders are changed for the formal implants in 8 weeks. In cases of postoperative radiation, there is a waiting period of 6 months after completion of the treatment, for device exchange.

## RESULTS

Skin reduction breast reconstruction using the Wise pattern with synchronous prepectoral placement of tissue expanders was performed on 6 breasts in 4 females (2 bilateral, and 2 unilateral). All reconstructions were performed by a single plastic surgeon, and the mastectomies were performed by a single breast surgeon.

The mean age was 55 (range, 40–73). Mean body mass index (BMI) was 37.6 (range, 32.3–43.0). One patient presented with grade 2 ptosis, whereas the other 3 had grade 3 ptosis. Mean mastectomy weight was 1,590 g (range, 1,246–2,294). Mean expander final fill volume was 510 ml. Mean fat volume used per reconstructed breast at the second stage was 31 ml.

One breast received postoperative radiation therapy without complications. There were 2 complications. One patient experienced superficial skin loss at the inverted-T edges requiring local wound care. Another patient experienced full-thickness skin necrosis at the inverted-T edges that required operative debridement and reclosure in the operating room. In this patient, intraoperative tissue assessment with indocyanine green was not performed at the time of the mastectomy, due to a documented Iodine allergy.

**Fig. 2.**
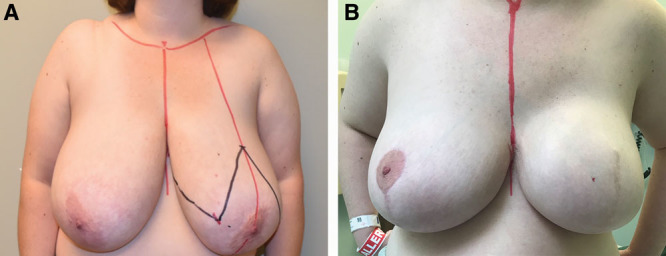
A, Preoperative frontal view of a 44-year-old female with grade 3 ptosis. B, Postoperative frontal image of a 44-year-old female obtained at 6 months follow-up after a left wise pattern mastectomy, and 2-stage breast reconstruction, with a right balancing breast reduction, and a left implant exchange with a 800 ml smooth round highly cohesive implant with extra projection.

There was no incidence of capsular contracture in nonradiated breasts, no device loss, and there were no systematic complications. In the 1 radiated breast, there was Baker Grade II capsular contracture at the time of the follow-up. All patients underwent expander-implant exchange and contralateral symmetry procedures (breast reduction) without complications (Table 1). The breast reduction technique that was used in the 2 cases was a Wise pattern with a superomedial pedicle at the time of the implant exchange (Fig. 2).

**Table 1.**
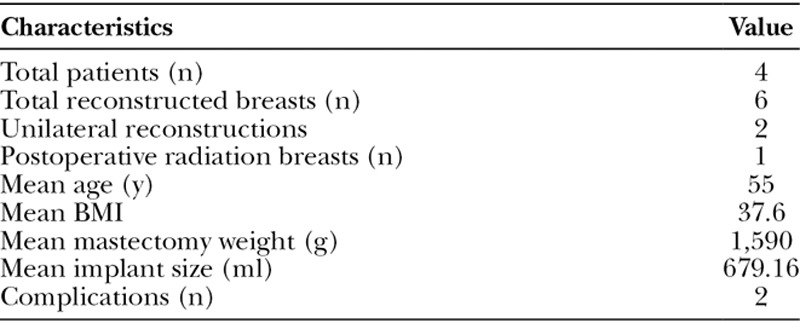
Patient Demographics and Perioperative Data

## DISCUSSION

Gabriel and Maxwell^3^ recently reported acceptable to good outcomes with 1 reconstructive failure out of 73 prepectoral expander/implant–based breast reconstructions in 39 patients with BMIs ≥ 35. Similarly, Caputo et al.^2^ previously described Wise Pattern direct-to-implant prepectoral reconstruction in 33 ptotic breasts. One breast presented with superficial necrosis, whereas 2 more breasts sustained full-thickness necrosis at the T-junction and were treated with excision and primary closure.^2^ The results from these previous studies are consistent with earlier published systematic reviews of both conventional and Wise Pattern skin-sparing mastectomy direct-to-implant and 2-stage reconstructions.^7,8^

The previously mentioned reviews both demonstrated increased risk of flap necrosis and implant loss associated with direct-to-implant procedures. Therefore, we incorporated a few key elements into our technique to mitigate this potential increased risk. First, flap viability and tension-free closure are the key tenets of this procedure. Intraoperative indocyanine green angiography was performed on all but 1 patient allowing for assessment of the flap in real time, and guiding the decision for immediate or delayed reconstruction.^9^ Regions of inadequate perfusion at the limits of the Wise Pattern are resected if immediate expander-based reconstruction is to be performed. Second, expanders are essential to achieving a tension-free closure with less vascular occlusion. Implant size in the setting of direct-to-implant may be limited as implant weight and projection may compromise vascularity.^8^ Furthermore, expanders allow more patient input with regard to final breast volume and also allow more predictable final results because refinements with skin resection or fat grafting for contouring can be performed during the implant exchange procedure.^3,7^ Prepectoral reconstruction and Wise pattern skin reduction can be performed in 1 stage, if the mastectomy flaps are of adequate thickness, and ideal implant selection can be performed, mainly in the context of bilateral reconstructions.

In anticipation of wound healing issues at the T-junction, there was inclusion of the deepithelialized dermal flap inferiorly to create a compound pocket.^2,10^ This flap provides support and militates expander loss should full-thickness necrosis occur at the T-junction because the Alloderm and expander will not be exposed.^3^ Necrosis can be excised and reclosed, or conservatively managed with debridement to the vascular dermal flap below and allowed to heal by secondary intention with dressing management. The flap in our study is also fenestrated to mitigate seroma formation between the Alloderm, dermal flap, and mastectomy skin flap.

**Fig. 3.**
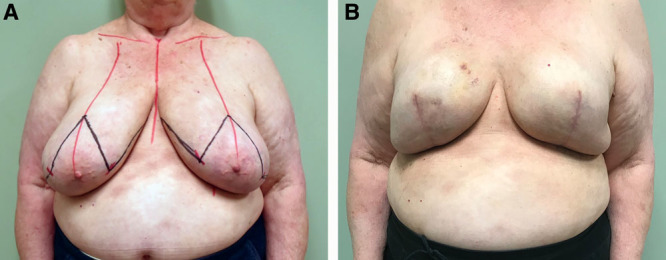
A, Preoperative frontal view of a 73-year-old female with grade 3 ptosis. B, Postoperative frontal image of a 73-year-old female obtained at 6 months follow-up after bilateral wise pattern mastectomy and 2-stage breast reconstruction with final placement of 605 ml smooth round highly cohesive implants with full projection.

This preliminary data suggest that skin reduction breast reconstruction with synchronous placement of the expander in the subcutaneous plane is a useful alternative with reasonable cosmetic results (Fig. 3), and a promising technique for females with large and ptotic breasts. Long-term studies with a larger number of patients are needed to better define the limitations of this newer surgical technique
